# Bilateral Macular Coloboma With Eccentric Fixation Confirmed by Microperimetry: A Case Report

**DOI:** 10.7759/cureus.92462

**Published:** 2025-09-16

**Authors:** Yoshinao Tamura, Makiko Wakuta, Ren Aoki, Masanori Mikuni, Fumiaki Higashijima, Takuya Yoshimoto, Manami Ohta, Kazutaka Yamamoto, Shinji Hirano, Kazuhiro Kimura

**Affiliations:** 1 Department of Ophthalmology, Yamaguchi University, Ube, JPN; 2 Department of Ophthalmology, Yamaguchi Prefectural Grand Medical Center, Hofu, JPN

**Keywords:** chorioretinal atrophy, eccentric fixation, macular coloboma, microperimetry, the stability of fixation

## Abstract

The purpose of this report is to describe a case of bilateral macular coloboma with eccentric fixation confirmed by microperimetry.

A 21-year-old Vietnamese woman presented with bilateral vision loss and was diagnosed with bilateral macular coloboma. Comprehensive ophthalmic examinations, including visual acuity testing, fundus examination, dynamic visual field testing, and microperimetry (MP-3, Nidek Co., Ltd., Gamagori, Japan), were performed to evaluate the macular lesions and fixation patterns. Blood tests were also conducted to investigate potential etiologies.

Visual acuity was found to be 20/100 in the right eye and 20/80 in the left eye, with exotropia but no nystagmus. Fundus examination revealed bilateral, round, well-defined depressions approximately four and three disc diameters in size in the macular areas, with choroidal atrophy, pigmentation, and linearization of retinal vessels. Dynamic visual field testing demonstrated paracentric dark spots corresponding to the lesions. Microperimetry showed unstable fixation located on the nasal side of the macular lesions in both eyes, with decreased retinal sensitivity in the affected areas. Blood tests were positive for cytomegalovirus immunoglobulin G (IgG).

This case presented a diagnostic challenge in distinguishing between developmental abnormality and inflammatory scarring due to a possible in utero infection. Microperimetry demonstrated eccentric fixation on the nasal side of the macular lesions, suggesting the patient likely established this fixation pattern from childhood, despite the unknown time of onset. This case highlights the value of microperimetry in evaluating fixation patterns in patients with bilateral macular coloboma.

## Introduction

Coloboma of the eye is a relatively rare, congenital anomaly occurring at a frequency of 0.5-2.2 cases per 10,000 live births [1]. The condition is thought to result from incomplete closure of the embryonic fissure during ocular development. Macular coloboma represents an even more infrequent clinical entity of particular importance due to its direct impact on central vision.

The etiology of macular coloboma remains incompletely understood, with evidence suggesting a multifactorial pathogenesis, including congenital developmental abnormalities, in utero infections, and post-natal inflammatory changes. Mann et al. proposed a classification system that divides macular colobomas into three distinct types: (1) pigmented macular coloboma, characterized by macular lesions covered with irregularly arranged dense pigmentation where large choroidal vessels are typically present; (2) non-pigmented macular coloboma, comprising round or oval punched-out patches devoid of choroidal and retinal vessels across the defective area; and (3) macular coloboma associated with abnormal vasculature, where retinal vessels may form atypical anastomoses with choroidal vessels within the lesion or extend anteriorly from the coloboma into the vitreous [2].

Previous clinical investigations have extensively utilized various imaging modalities to characterize macular coloboma. Optical coherence tomography (OCT) typically reveals a crater-like depression in the macula with atrophic neurosensory retina and absence of retinal pigment epithelium (RPE) and choroid within the lesion, as described by Gunderia et al. [3]. Fluorescein angiography has been employed to detect vascular abnormalities, while visual field testing has helped identify functional defects. However, there remains a notable paucity of detailed studies examining visual field characteristics, fixation position, and stability in patients with macular coloboma.

In this case report, we provide novel insights into the mechanisms of visual adaptation in macular coloboma by comprehensively evaluating visual function in a patient with bilateral macular coloboma, with particular emphasis on detailed analysis of fixation patterns using microperimetry.

## Case presentation

The patient was a 21-year-old Vietnamese national. During a physical examination, she was found to have decreased visual acuity in both eyes and retinal choroidal atrophy in the macular area. She was referred to our department for a close examination. There was no history of ophthalmologic or systemic diseases, nor a family history of similar diseases.

**Figure 1:**
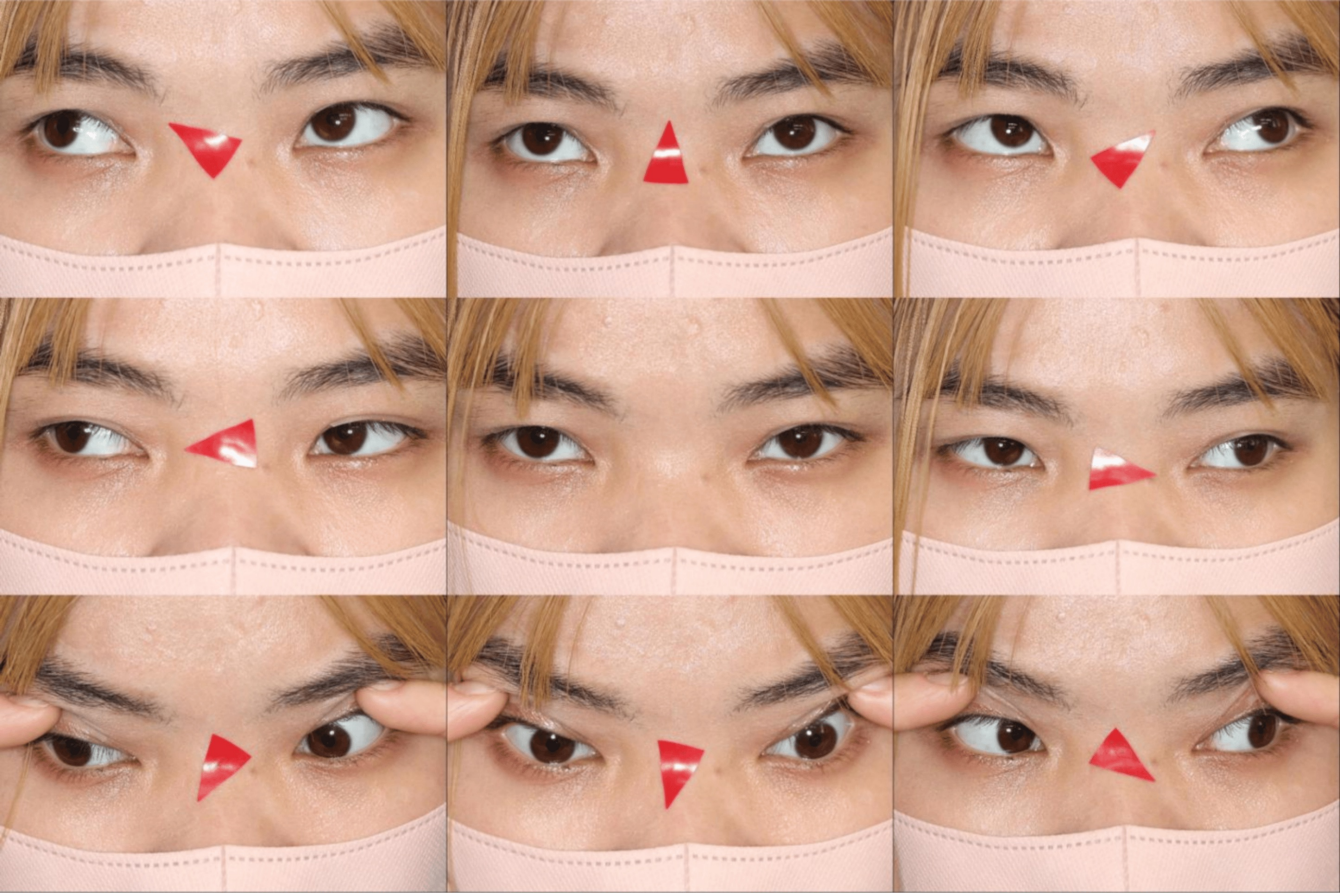
Nine-gaze photographs of her strabismus Exotropia was observed, but no limitation of ocular motility was noted.

At the time of initial examination, her visual acuity was 20/100 in the right eye and 20/80 in the left eye without correction, both uncorrectable. The axial length measured by the IOL Master (Carl Zeiss Meditec AG, Jena, Germany) was 22.62 mm in the right eye and 23.31 mm in the left eye. However, as fixation did not correspond to the fovea in this case, these measurements were deemed less reliable. Intraocular pressure was 10 mmHg in both eyes. The dominant eye was the left eye. The left eye was the fixating eye, and the right eye had 20 prism diopters of exotropia in the near and 18 prismatic dioptric exotropia in the far eye (Figure 1).

There were no abnormal findings in the ocular movements of either eye and no nystagmus. There were also no abnormal findings in the anterior areas of both eyes.

**Figure 2:**
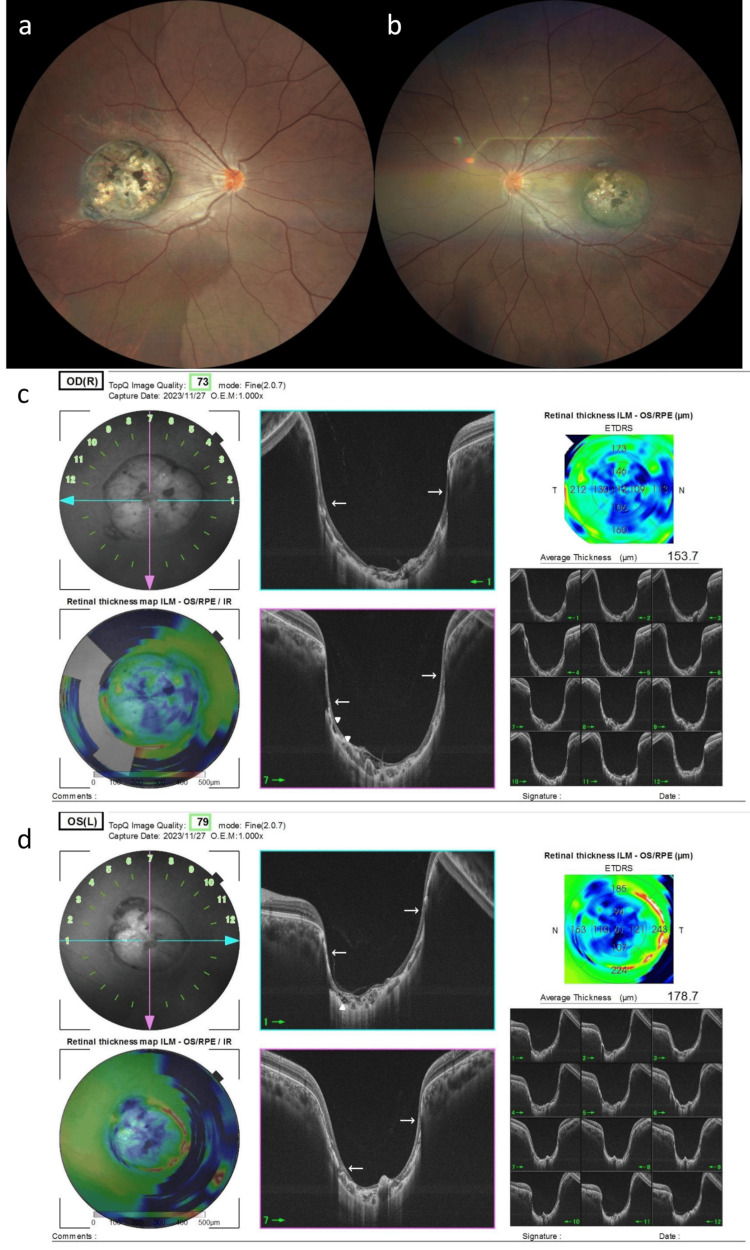
Fundus photographs of the case at the first visit (a) Fundus of the right eye. (b) Fundus of the left eye. (c) OCT of the right eye. (d) OCT of the left eye. A clearly demarcated, pigmented, depressed lesion measuring approximately four disc diameters in the right macula and three disc diameters in the left macula was observed, and the choroidal vessels and sclera were visible. A continuous retinal pigment epithelium layer (white arrow), a hollow choroid (white arrowhead), and an atrophic retinal structure are observed.

Fundus examination revealed well-defined circular depressions measuring approximately four disc diameters in the right macula and three disc diameters in the left macula, accompanied by choroidal defects with pigmentation and retinal atrophy in the same areas (Figures 2a, 2b).

**Figure 3:**
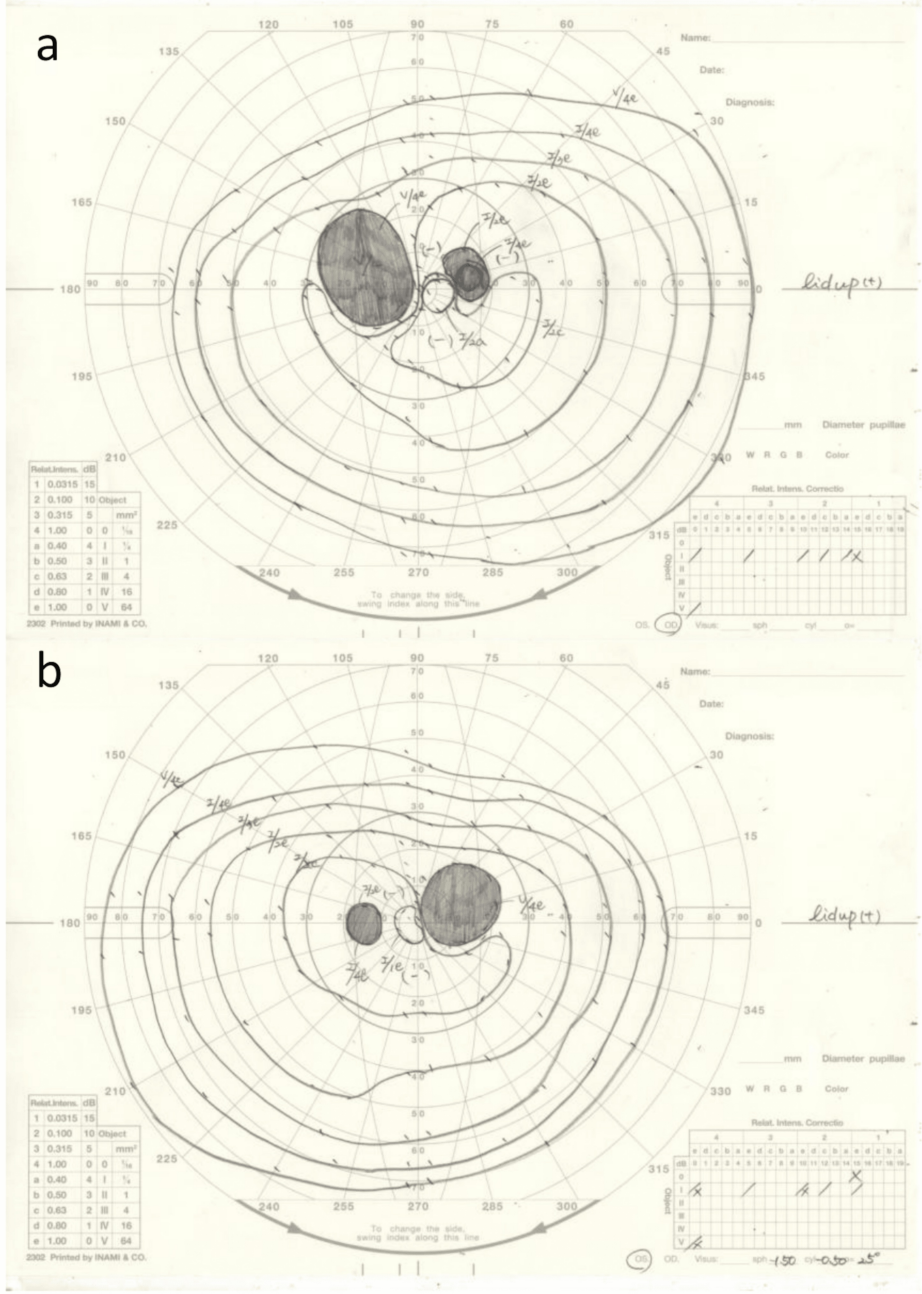
Dynamic visual field testing of the case (a) Dynamic visual field testing of the right eye. (b) Dynamic visual field testing of the left eye. A paracentric dark spot is present in an area consistent with the lesion.

OCT revealed a crater-like depression in the macula of both eyes, as well as choroidal defects and retinal atrophy in the same area (Figures 2c, 2d). Dynamic visual field testing showed a paracentral dark spot in the central nasal area in both eyes, with a size corresponding to the lesions (Figures 3a, 3b).

**Figure 4:**
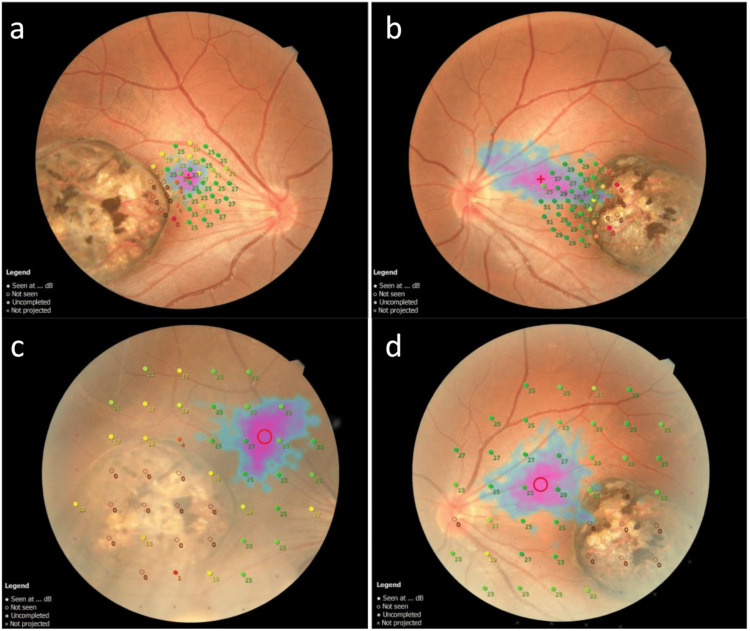
Microperimetry of the case Microperimetry of the right eye (a) and left eye (b) at 1 month after the initial consultation, and of the right eye (c) and left eye (d) at 15 months. At the time of the initial examination, unstable fixations were observed in the normal retina outside the macular lesion, accompanied by reduced sensitivity within the lesion; a reexamination at 15 months showed almost the same findings.

Microperimetry (MP-3, Nidek Co., Ltd., Gamagori, Japan) at one month after the initial consultation also showed reduced sensitivity to the lesion in both eyes, and unstable fixation was detected in the normal retina nasal to the macular lesion (Figures 4a, 4b).

An electroretinogram (ERG) showed almost normal waveform shape, with normal amplitude and latency. Multifocal ERG showed a weakening of the waveform in the nasal visual field, including the area of macular atrophy.

Blood tests showed no abnormalities in both biochemistry and hematology: treponema pallidum antibody negative, rapid plasma reagin test negative, toxoplasma antibody immunoglobulin Ig (IgG) and immunoglobulin M (IgM) both negative, tuberculosis-specific interferon-γ negative, herpes simplex virus antibody IgG positive, IgM negative, varicella zoster virus antibody IgG positive, IgM negative, cytomegalovirus antibody IgG positive, IgM negative, human T-lymphotropic virus-1 antibody negative, antinuclear antibody negative, and Rheumatoid factor negative.

Based on fundus findings and OCT showing depressed lesions with chorioretinal atrophy in both maculae and no other findings such as causative infection, a diagnosis of bilateral macular coloboma was made.

At 15 months, there were no changes in visual acuity or other examination findings compared to the initial visit. No progression of fundus lesions or new findings, such as retinal detachment, were observed, and therefore no treatment was administered. MP-3 microperimetry was re-evaluated at the same time, showing decreased sensitivity in the lesion of both eyes, with fixation remaining unstable and unchanged (Figures 4c, 4d).

## Discussion

In the present study, we experienced a rare case of bilateral macular coloboma. Despite fundus findings and OCT showing depressed lesions with choroidal loss and retinal atrophy in the macular areas of both eyes, she had mild subjective symptoms, and her visual acuity was 20/100 in the right eye and 20/80 in the left eye, which was not extremely low. The microperimetry showed an unstable fixation in the nasal normal retina from the location of the fovea centralis. This is the first report of a shift of the fixation in a case of bilateral macular coloboma.

Coloboma is a condition in which normal tissue in or around the eye is defective from birth. This disease is a tissue defect during development, and depending on the site, coloboma of the eyelid, lens, macula, optic nerve, uvea, and chorioretina have been reported. Known causative genes include RARB, BMP7, TFAP2A, and CHD7. Macular coloboma is characterized by a well-defined, depressed, atrophic lesion of approximately 3-6 disc diameters in the center of the macula [4]. It is also a rare disease with an incidence of 0.5-0.7 per 1000 live births [5]. According to previous reports, visual acuity in macular coloboma ranges from counting fingers to 20/25 [4]. There are a variety of diseases that must be differentiated from macular coloboma, including Leber congenital amaurosis, central choroidal atrophy, progressive pyramidal dystrophy, posterior staphyloma, and macular scarring due to infectious choroiditis. In particular, postinflammatory scarring due to Toxoplasma infection is similar to the scarring of macular coloboma and is difficult to differentiate [6]. In the present case, anti-Toxoplasma antibodies were negative for both IgG and IgM.

In this case, microperimetry was used to evaluate the fixation. Microperimetry is the general term for a testing method that measures local retinal sensitivity by presenting a light stimulus to the fundus. It can evaluate the location and stability of fixation in patients with macular disease [7]. In the present case, the fixation was shifted to the nasal side of the original macular area, and an unstable fixation was observed. The fixation of both eyes was unstable in both tests, but because the fixation of the left eye was the same in both tests, it was believed that the left eye had better visual acuity than the right eye, which had an unstable fixation and was functioning as the dominant eye. When the visual function of the macula is severely impaired by macular disease, central fixation is no longer possible, and fixation shifts off-center. In the process, patients without established eccentric fixation tend to try to see things in the invisible central region [8]. The fixation in that eye is considered unstable and shifting. According to previous reports in other diseases, there were cases in patients with age-related macular degeneration in which the fixation moved with an oscillating motion across the boundary between the atrophic area and the normal retina as macular atrophy expanded, and cases in which a false fixation was initially observed within the atrophic area, but over time, it became eccentric and visual acuity improved [8]. Also, in a report of 29 eyes of patients with macular dystrophy, the fixation was located within the atrophic lesion in 19 eyes and outside the atrophic lesion in 10 eyes [9]. In cases with migrating fixation, it was reported that many of the cases had migrated above the central fossa.

In this case, microperimetry was used for macular coloboma to assess retinal sensitivity and fixation in the lesion and outside the lesion. Retinal sensitivity in the lesion was reduced, and the fixation was located outside the atrophic lesion, with eccentric fixation. The fixation was also nasally deviated. In the report of macular dystrophy presented in the previous paragraph, the process of migration of the fixation and the time since diagnosis were not specified in the group in which the fixation was present within the atrophic lesion and the group in which it was present outside the atrophic lesion. Unlike diseases that occur later in life, macular coloboma is a congenital condition, and eccentric fixation may have been established from early childhood. On the other hand, some studies have shown that eccentric fixation training using microperimetry in patients with age-related macular degeneration and geographic atrophy led to improvements in fixation stability, visual acuity, and reading speed [10]. Although unstable fixation was observed in the present case, future eccentric fixation training with microperimetry may improve fixation stability and enhance visual function.

## Conclusions

We recently experienced a rare case of bilateral macular coloboma. In this case, we evaluated the fixation in bilateral macular coloboma and found an unstable fixation outside the lesion. The results of microperimetry in this case suggested that even in cases with atrophy or degeneration in the macula in both eyes, the fixation may move outside the fovea, and vision may be obtained through eccentric fixation.
